# IP-10 and MIG are sensitive markers of early virological response to HIV-1 integrase inhibitors

**DOI:** 10.3389/fimmu.2023.1257725

**Published:** 2023-10-18

**Authors:** Hortensia Álvarez, Alicia Gutiérrez-Valencia, Ana Mariño, Abraham Saborido-Alconchel, Beatriz Calderón-Cruz, Alexandre Pérez-González, Jacobo Alonso-Domínguez, Inés Martínez-Barros, María Gallego-Rodríguez, Santiago Moreno, Teresa Aldamiz, Marta Montero-Alonso, Enrique Bernal, Carlos Galera, Josep M. Llibre, Eva Poveda

**Affiliations:** ^1^Infectious Diseases Unit, Department of Internal Medicine, Complexo Hospitalario Universitario de Ferrol, Servicio Galego de Saúde (SERGAS)-A Coruña, A Coruña, Spain; ^2^Department of Biochemistry, Genetics and Immunology, Universidade de Vigo, Vigo, Spain; ^3^Clinical Unit of Infectious Diseases and Microbiology, Institute of Biomedicine of Seville (IBiS), Virgen del Rocío University Hospital, Spanish National Research Council (CSIC), University of Seville, Seville, Spain; ^4^Galicia Sur Health Research Institute (IIS Galicia Sur)-Complexo Hospitalario Universitario de Vigo, Servicio Galego de Saúde-Universidade de Vigo (SERGAS-U, Vigo), Vigo, Spain; ^5^Infectious Diseases Department, Hospital Universitario Ramón y Cajal, Madrid, Spain; ^6^Infectious Diseases Unit, Hospital General Universitario Gregorio Marañón, Madrid, Spain; ^7^Infectious Diseases Unit, Hospital Universitario y Politécnico La Fe, Valencia, Spain; ^8^Infectious Diseases Unit, Hospital General Universitario Reina Sofía, Murcia, Spain; ^9^Internal Medicine Department, Hospital Universitario Virgen de la Arrixaca, Murcia, Spain; ^10^Infectious Diseases Division and Fight Infections Foundation, University Hospital Germans Trias i Pujol, Badalona, Barcelona, Spain

**Keywords:** antiretroviral treatment, IP-10, MIG, biomarkers, HIV-1 viral decay

## Abstract

**Background:**

Interferon-inducible protein-10 (IP-10) and monokine induced by interferon-gamma (MIG) are chemokines recognized as inflammatory biomarkers during HIV-1 infection. We assessed their early and long-term dynamics after initiation of antiretroviral treatment (ART).

**Methods:**

Persons with HIV-1 (PWH) aged>18 years starting their first ART in 2015-2021 in a prospective cohort (n=73) were included. IP-10 and MIG plasma levels were quantified using a multiplexed bead-based assay.

**Results:**

IP-10 and MIG plasma levels showed a significant and consistent reduction following ART (80% integrase inhibitor [INSTI]-based) initiation, starting at day 20 and maintained throughout the study period (48 months), paralleling the HIV-1 RNA decay and CD4+ count recovery (p<0·001). At baseline, PWH≥ 50 years, CDC stage C and CD4+ count<350cells/mm^3^ had higher levels of IP-10 (p=0·022, p=0·001 and p=0·002, respectively) and MIG (p<0·001, p=0·024 and p=0·069, respectively). All of them matched their counterparts several months following ART initiation. MIG levels showed a greater decrease at day 10 in those treated with INSTI (p=0·038). Low-level HIV-1 viremia did not impact MIG or IP-10 levels.

**Conclusion:**

Plasma IP-10 and MIG showed an early significant decline following ART initiation, with greater early declines in MIG levels in INSTI-based regimens. These findings suggest a strong impact of HIV-1 viremia on IP-10 and MIG levels.

## Introduction

Chronic immune activation underlies multiple pathways along the course of HIV-1 infection, and a persistent inflammatory state is considered a driver of morbidity (cardiovascular, neurocognitive, and pulmonary diseases, neoplasia, genomic instability, immunosenescence, and premature aging) and mortality (1, 2). Interferon-inducible protein-10 (IP-10), also known as C-X-C motif chemokine ligand (CXCL)-10, and monokine induced by interferon-gamma (MIG), also known as CXCL-9, are pro-inflammatory chemokines originated from circulating myeloid cells (mainly monocytes and dendritic cells) and lymphoid organs (largely from the small intestine) in response to interferon. Both cytokines are ligands for the C-X-C chemokine receptor (CXCR)3, which is highly expressed on memory CD4+ T cells, a major cell target for HIV-1 (3). In addition, IP-10 can recruit susceptible T cells to lymph nodes, contributing to immune activation and dysfunction as well as helping to establish viral reservoirs (4–6). These chemokines have been previously explored in persons with HIV-1 (PWH) both in early and chronic infection (4–15). IP-10 and MIG are upregulated early after primary HIV-1 infection. IP-10 begins to increase on approximately day 6 after the first positive viral load assessment, which is a more rapid response than that observed for MIG (levels increase one month after infection) (7, 8, 11, 12). Indeed, IP-10 has been assessed as an accurate screening tool to detect acute HIV-1 infection in resource-limited settings (8). Elevated IP-10 levels are associated with rapid loss of CD4+ T cells upon HIV-1 infection and faster disease progression, even in individuals with CD4+ T cell count above 500 cells/mm^3^ (4, 11, 12, 16). Interestingly, we previously observed higher IP-10 and MIG levels in elite controllers who later lost viral control compared with persistent controllers, highlighting the interest of these cytokines as potential biomarkers to predict loss of viremic control (17).

After HIV-1 infection, a strong positive correlation of IP-10 with HIV-1 RNA and a negative correlation with CD4+ T cell count has been reported (4, 18). In turn, after antiretroviral treatment (ART) initiation, there is significant parallelism of plasma IP-10 and MIG levels decrease with HIV-1-RNA suppression and CD4 cell count recovery (15), in such a way that persistent levels of IP-10 can be associated with a CD4+ T cell count that fails to recover (19). However, no studies have assessed their dynamics after treatment initiation with integrase inhibitors.

We aimed to assess the dynamics in plasma IP-10 and MIG levels throughout the study period in a well-characterised cohort of PWH initiating current antiretroviral regimens including integrase inhibitors. Analyses were focused on particular scenarios of interest, stratifying according to age, AIDS-defining events, and CD4+ T cell count at baseline and HIV-1 RNA at month 12 after ART initiation.

## Materials and methods

### Samples

We used frozen plasma samples stored at -80°C that were prospectively collected from 32 HIV-1-infected treatment-naïve individuals in clinical follow-up at the Spanish HIV-1/AIDS Research Network Cohort (CoRIS), a nationwide multicentre cohort launched in 2004, including individuals with confirmed HIV-1 infection who were recruited in 46 HIV-1 care units of the Spanish Public Health System. A complete description of CoRIS has been published previously (20). Samples were provided by the HIV-1 BioBank integrated into the Spanish AIDS Research Network (RIS).

In addition, we used plasma samples that were prospectively collected from 41 HIV-1-infected treatment-naïve individuals in clinical follow-up at the Complexo Hospitalario Universitario de Ferrol, A Coruña, Galicia, Spain (Ferrol Cohort), who started ART in April 2017. Blood samples were processed at the Galicia Sur Health Research Institute to obtain plasma and peripheral blood mononuclear cells and stored at the Biobank of Vigo for subsequent analysis. Overall, 73 individuals aged > 18 years and who initiated ART from February 2015 to July 2021 were included in the current analysis according to their sample availability in the time points of interest for plasma cytokine measurements.

### Ethical considerations

All individuals participating in the study gave their informed consent and protocols were approved by institutional ethical committees.

### Chemokine measurement

We assessed changes in plasma chemokine profiles (IP-10 and MIG) pre- and post-ART initiation in the study population, at the following sampling time points: baseline and months 6 and 12 after ART initiation. For the Ferrol cohort, it was possible to assess earlier time points including day 10, day 20, and months 1 and 3 after ART initiation. In turn, within the CoRIS cohort, it was also possible to evaluate longer time points including months 24, 36, and 48 after ART initiation. Plasma chemokine changes were compared with HIV-1 RNA and CD4+-T cell dynamics. Several particular scenarios were assessed: the subset of individuals aged ≥50 or <50 years, baseline CDC category (defining C as having an AIDS-defining event, at HIV-1 diagnosis), baseline CD4+ T count <350 cells/mm^3^ vs. CD4+ T count ≥350 cells/mm^3^, and the initial antiretroviral (ARV) regimen (two nucleos[t]ide reverse transcriptase inhibitors [NRTIs] plus a third drug including boosted-protease inhibitors [b-PI] or integrase strand transfer inhibitors [INSTI]).

In addition, within the Ferrol cohort, we were able to explore the IP-10 and MIG levels in individuals with low-level viremia (defined as HIV-1 RNA 20-199 copies/mL), and target detected (TD) or not detected (TND) HIV-1 RNA below 20 copies/mL, at month 12 after ART initiation.

Determinations of IP-10 and MIG (pg/mL) were performed using a multiplex bead-based immunoassay (MILLIPLEX^®^ MAP human high-sensitivity T cell magnetic bead panel; Merck EMD Millipore, Billerica, MA, US).

### Statistical analysis

Statistical significance was determined using Friedman, Wilcoxon, Kruskal-Wallis, Spearman´s correlation, and Mann-Whitney tests. All statistical analyses were two-tailed, with an alpha of 0.95; values were considered significant at p <0·05. In the multiple comparisons, all p-values were adjusted using the Bonferroni correction. Analyses were performed using SPSS, IBM, version 19.0.

Descriptive statistics were calculated based on their distributions among the quantitative variables. The normality of these variables was calculated with the Shapiro-Wilk test. Regarding the categorical variables, frequencies and proportions were determined.

The Friedman test was used to estimate differences in medians and interquartile range (IQR) among continuous variables (cytokines, CD4, and HIV-1 RNA plasma concentrations) at different time points. We used the Mann-Whitney U test to compare the median concentrations of plasma determinations among groups. In addition, we utilized Spearman’s correlation coefficient to correlate quantitative variables.

Finally, we used a logistic regression model to assess the impact of multiple baseline predictor variables on high concentrations of plasma IP-10 and MIG at month 12 after ART initiation, defined as >370pg/mL and >955pg/mL, respectively, based on cutoffs used in a previous analysis (15). Values were expressed as adjusted odds ratios (aORs) and 95% confidence intervals (CIs). Baseline variables were defined *a priori*, before the ART initiation date. Models were adjusted for sex, age, comorbidities, CD4 cell count <350 cells/mm^3^ at ART initiation, and CDC stage C at HIV infection.

## Results

### Characteristics of the study population

The baseline characteristics of participants are shown in **Table 1**. Overall, we included 73 ART-naïve individuals, of which 83.6% were men, 80.8% were individuals from Spain, and 60.2% were men who had sex with other men or declared themselves as being bisexual. The median age was 37 (IQR 31-49) years and 18 (24·6%) individuals were aged 50 years or older. The median CD4+ count was 332 (IQR 149-672) cells/mm^3^, 36 (49.3%) participants had CD4+ counts <350 cells/mm^3^, 38.3% had HIV-1 RNA >100,000 copies/mL at ART initiation, and 80.8% had initiated an INSTI-based regimen.

**Table 1:** Demographic and clinical characteristics of the study cohort.

	Overall n=73	Ferrol cohort n= 41	CoRIS cohort n=32
Sex			
**Male, n (%)**	**61 (83·6)**	**32 (78·0)**	**29 (90·6)**
**Age (years), median (IQR)** **≥50 years, n (%)**	**37 (31-49)** **18 (24·6)**	**42 (32-52)** **14 (34·1)**	**33 (30-43)** **4 (12·5)**
**BMI (kg/m^2^), median (IQR)**	**24·2 (20·5-28·1)**	**24·3 (20·2-28·3)**	**23·3 (21·4-27·2)**
Region of origin			
**Spain, n (%)** **Other, n (%)**	**59 (80·8)** **14 (19·2)**	**36 (87·8)** **5 (12·1)**	**23 (71·9)** **9 (28·1)**
Current drug use, at ART initiation			
**Tobacco, n (%)** **Alcohol (> 30 g/day), n (%)** **Other, n (%)**	**31 (42·5)** **5 (6·8)** **8 (10·9)**	**21 (51·2)** **5 (12·2)** **8 (19·5)**	**10 (31·3)** **0** **0**
Comorbidities			
**Yes, n (%)**	**20 (27·4)**	^a^15 (36·6)	**5 (15·6)**
HIV transmission route			
**MSM/bisexual, n (%)** **Heterosexual, n (%)** **IDU, n (%)**	**44 (60·2)** **24 (32·9)** **2 (2·7)**	**22 (53·6)** **17 (41·5)** **2 (4·9)**	**22 (68·8)** **7 (21·9)** **0**
**HCV-coinfection (detected plasma RNA), n (%)**	**1 (1·3)**	**1 (2·4)**	**0**
CDC Category (1993)			
**A, n (%)** **B, n (%)** **C, n (%)** **Unknown, n (%)**	**54 (74·0)** **6 (8·2)** **11 (15·1)** **2 (2·7)**	**28 (68·3)** **4 (9·7)** ^b^9 (22·0) **0**	**26 (81·3)** **2 (6·3)** **2 (6·3)** **2 (6·3)**
Initial ART			
**Classes** **Two NRTI plus INSTI, n (%)** **Two NRTI plus b-PI, n (%)** **Two NRTI plus NNRTI, n (%)** **Other** **ART regimen (the most frequent)** **BIC/FTC/TAF, n (%)** **DTG/3TC/ABC, n (%)** **EVG/c/FTC/TAF, n (%)** **TDF/FTC+DRV/c, n (%)**	**59 (80·8)** **7 (9·6)** **2 (2·7)** **5 (6·8)** **15 (20·5)** **19 (26·0)** **5 (6·8)** **5 (6·8)**	**36 (87·8)** **5 (12·2)** **0** **0** **15 (36·6)** **11 (26·8)** **3 (7·3)** **3 (7·3)**	**23 (71·9)** **2 (6·3)** **2 (6·3)** **5 (15·6)** **0** **8 (25·0)** **2 (6·3)** **2 (6·3)**
^c^Baseline CD4 + T cells/mm^3^, median (IQR)	**332 (149-672)**	**323 (54-663)**	**376 (222-716)**
^c^Baseline CD4+ T < 350 cells/mm^3^, n (%)	**36 (49·3)**	**23 (56·0)**	**13 (40·0)**
^c^Baseline CD4+T/CD8+ T cell ratio; median (IQR)	**0·35 (0·15-0·59)**	**0·29 (0·11-0·59)**	**0·43 (0·15-0·62)**
^c^Baseline HIV-1 RNA, copies/mL; median (IQR)	**58,100 (13,966-338,000)**	**87,600 (19,400-552,500)**	**29,192 (2,636-98,690)**
^c^Baseline HIIV-1 RNA >100,000 copies/mL, n (%)	**28 (38·3)**	**20 (48·7)**	**8 (25·0)**
HIV genotype			
**B, n (%)** **Non-B, n (%)**	**-** **-**	**19 (46·3)** **22 (53·6)** C 1 F1 21	- -

BMI, body mass index; ART, antiretroviral treatment; IQR, interquartile range; MSM, men who have sex with men; IDU, injected drug user; HCV, hepatitis C virus; NRTI, nucleos(t)ide reverse transcriptase inhibitor; b-PI, boosted protease inhibitor; INSTI, integrase strand transfer inhibitor; TDF, tenofovir disoproxil fumarate; ART, antiretroviral treatment; TAF, tenofovir alafenamide; ABC, abacavir; 3TC, lamivudine; FTC, emtricitabine; DRV/c, darunavir/cobicistat; EVG/c, elvitegravir/cobicistat; DTG, dolutegravir; BIC, bictegravir.

a Comorbidities: cardiovascular (6), neuropsychiatric (6), respiratory (5), renal (2).

b CDC stage C: AIDS-defining events included most but not only opportunistic infections: disseminated tuberculosis (1), extrapulmonary crytococcosis (2), Pneumocystis jirovecii pneumonia (2), protracted genital herpetic ulcer (1), disseminated Mycobacterium avium complex infection (1), non-Hodgkin lymphoma (1), wasting syndrome (4).

c Baseline CD4+ count and HIV-1 RNA were defined as the last measurement before the ART initiation date.

### IP-10 and MIG dynamics after ART initiation

**Figure 1:**
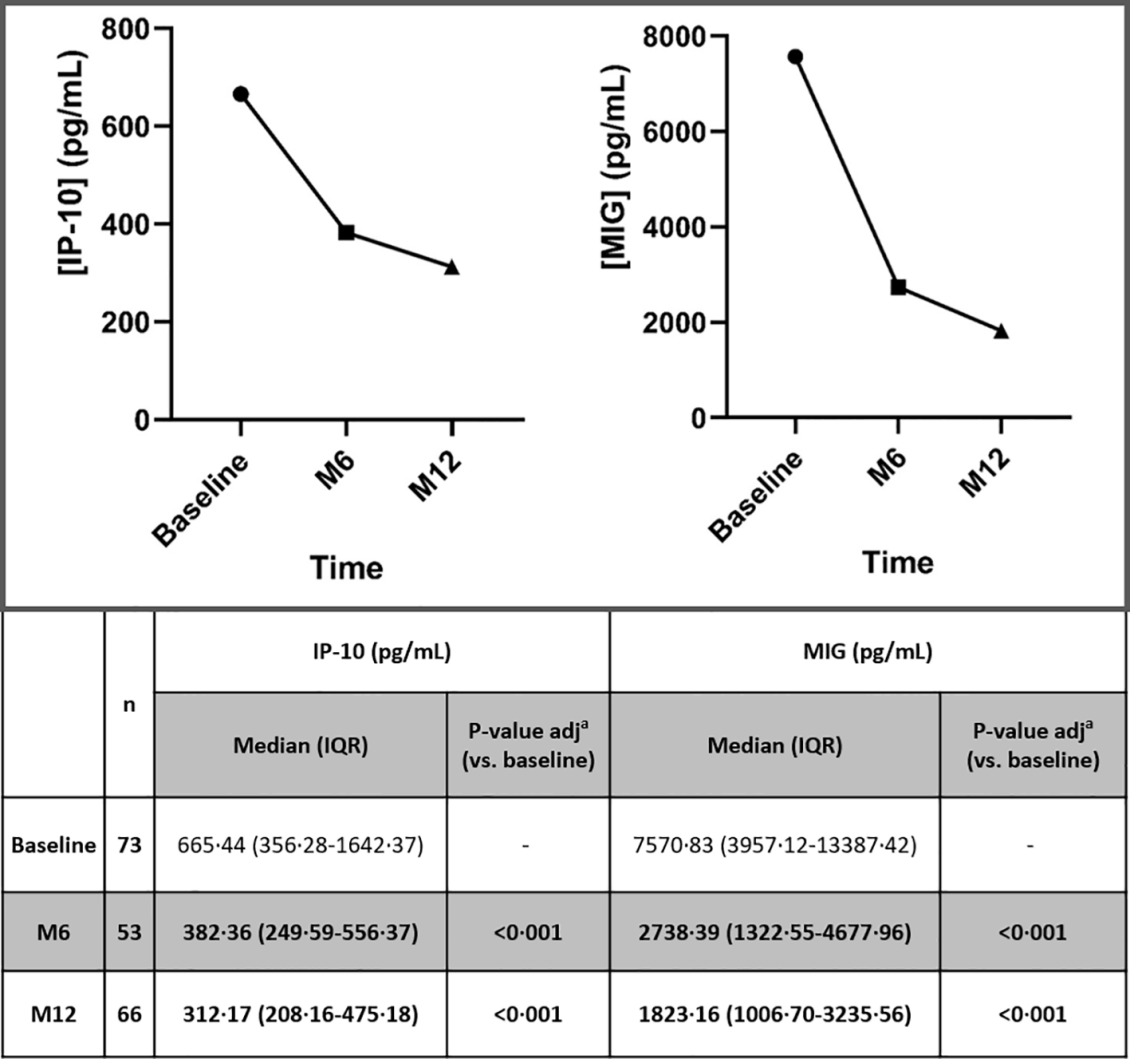
Dynamic changes in IP-10 and MIG levels throughout 12 months, after ART initiation. IP-10, Interferon-inducible protein-10; MIG, Monokine induced by interferon-gamma; ART, antiretroviral treatment; n, number of participants; M6, month 6; M12, month 12; IQR, interquartile range. Statistically significant values of variables are highlighted in bold. IP-10 and MIG plasma levels are represented as plasma concentrations median values. ^a^p-value adjusted by the Bonferroni correction.

**Figure 2:**
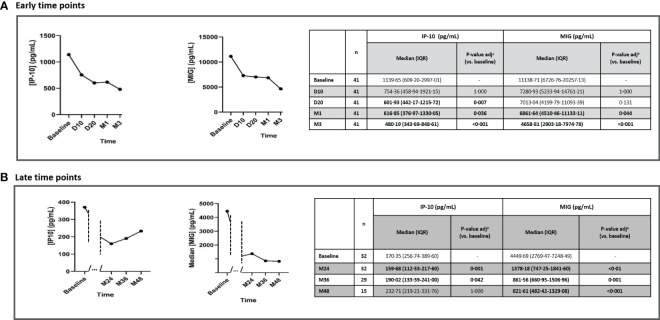
Dynamic changes in IP-10 and MIG levels throughout early time points **(A)** and late time points **(B)**, after ART initiation. IP-10, Interferon-inducible protein-10; MIG, Monokine induced by interferon-gamma; n, number of participants; ART, antiretroviral treatment; D10, day 10; D20, day 20; M1, month 1; M3, month 3; M24, month 24; M36, month 36; M48, month 48; IQR, interquartile range. Statistically significant values of variables are highlighted in bold. IP-10 and MIG plasma levels are represented as plasma concentrations median values. ^a^p-value adjusted by the Bonferroni correction.

IP-10 and MIG plasma levels showed a significant and consistent decrease vs. baseline throughout the study period following ART initiation (p<0·001) (**Figures 1**, **2**). This decline was consistently and significantly seen in early time points (day 20) for IP-10 (p=0·007) (**Figure 2A**) and throughout 12 months for both IP-10 and MIG (p<0·001) (**Figure 1**) after ART initiation. In addition, this trend was confirmed throughout 36 months (for both IP-10 and MIG, p=0·042 and p=0·001, respectively) but only for MIG (p<0·001) at 48 months (for IP-10, p=1·000 at 48 months) (**Figure 2B**).

The earlier decay in HIV-1 RNA (month 1, p<0·001) preceded the CD4 cell count increase (month 6, p<0·001) (**Table S1**).

**Figure 3:**
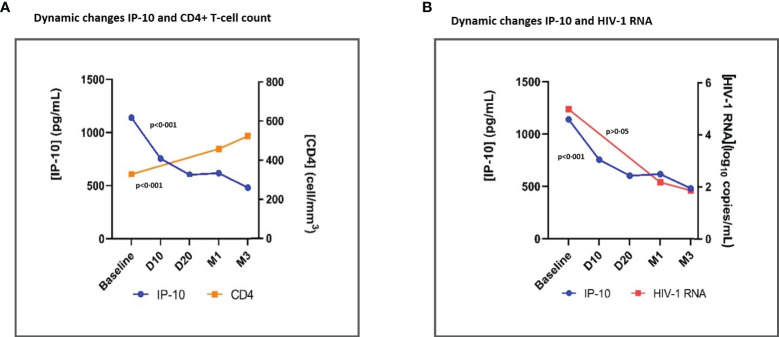
Dynamic changes in IP-10 levels and CD4+ T-cell count **(A)** and dynamic changes in IP-10 plasma levels and HIV-1 RNA **(B)**, throughout early time points after ART initiation. IP-10, Interferon-inducible protein-10; ART, antiretroviral treatment; D10, day 10; D20, day 20; M1, month 1. Global tendency (throughout all time points represented in the graph) p-value, calculated with the Friedman Test. IP-10, CD4+ T cell count and HIV-1 RNA plasma levels are represented as plasma concentrations median values.

**Figure 4:**
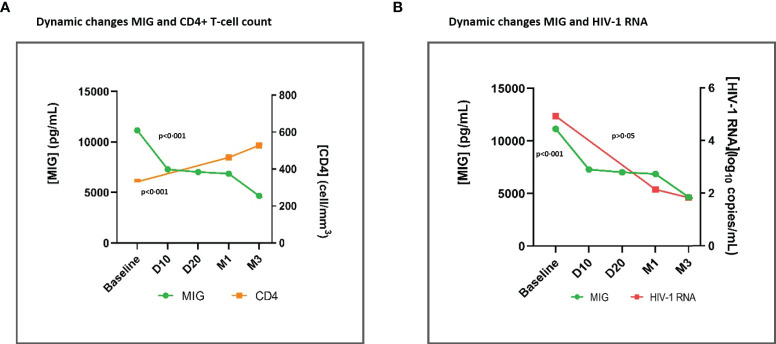
Dynamic changes in MIG levels and CD4+ T-cell count **(A)** and dynamic changes in MIG levels and HIV-1 RNA **(B)**, throughout early time points after ART initiation. MIG, Monokine induced by interferon-gamma;ART, antiretroviral treatment; D10, day 10; D20, day 20; M1, month 1. Global tendency (throughout all time points represented in the graph) p-value, calculated with the Friedman Test. MIG, CD4+ Tcell count, and HIV-1 RNA plasma levels are represented as plasma concentrations median values.

Overall, there was a significant (p<0·001) parallelism of IP-10 and MIG decrease with CD4+ count recovery (p<0·001) and the HIV-1-RNA decay (p<0·001) at month 12 (**Figure S1**) and in early time points specifically (**Figures 3**, **4**). Moreover, we observed a positive correlation between IP-10 and MIG plasma levels and HIV-1 RNA, which was more concordant in early time points. Likewise, there was a negative correlation between IP-10 and MIG plasma levels and CD4+ count, which was both overall throughout 12 months (**Figures S2**, **S3**) and in early time points (**Figures S4**, **S5**).

### Impact of age, baseline CDC stage, CD4+ count, and initial third drug of ART on IP-10 and MIG levels

**Figure 5:**
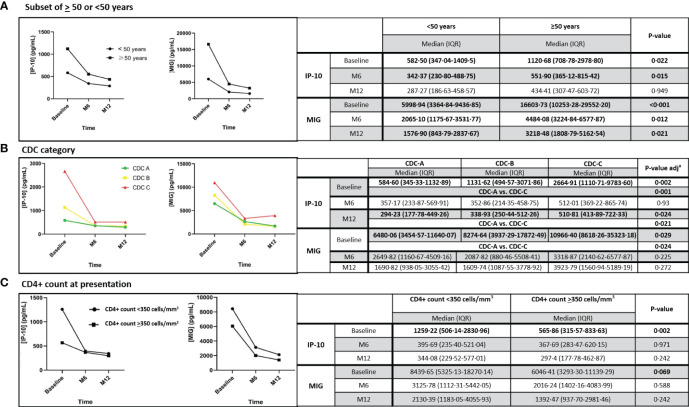
Dynamic changes in IP-10 and MIG levels, in the subset of individuals aged ≥ 50 or <50 years **(A)**, according to the CDC category **(B)** and to the CD4+ count **(C)** at ART initiation, throughout 12 months after ART initiation. IP-10, Interferon-inducible protein-10; MIG, Monokine induced by interferon-gamma; M6, month 6; M12, month 12; IQR, interquartile range. Statistically significant values of variables are highlighted in bold. IP-10 and MIG plasma levels are represented as plasma concentrations median values. In **Figure 5B**, ^a^p-value is adjusted by the Bonferroni correction.

At baseline, PWH aged ≥ 50 years had significantly higher levels of IP-10 (p=0·022) and MIG (p<0.001) than PWH aged <50 years. Upon ART initiation, IP-10 levels in PWH aged ≥50 years showed a sharp reduction and at month 12, IP-10 levels matched PWH aged <50 years. Similarly, MIG levels showed a sharp decrease in PWH aged ≥50 years, but the late decline was slower, and at month 12, differences were still significant vs. PWH aged <50 years (p=0·021) (**Figure 5A**). However, it´s worth noting that age at ART initiation ≥50 years was not associated with high levels of IP-10 and MIG at month 12 after ART initiation, in the multivariate analysis (**Table 2**).

**Table 2:** Logistic regression analysis (multivariate) of factors associated with high levels of IP-10 and MIG levels at month 12 after ART initiation.

	Plasma IP-10 >370pg/mL at month 12		Plasma MIG >955pg/mL at month 12	
	Adjusted OR (95% CI)	P-value	Adjusted OR (95% CI)	P-value
Sex				
**Female** **Male**	1·48 (0·34-6·36) 1·0 (Ref.)	0·596	1·28 (0·22-7·43) 1·0 (Ref.)	0·777
Age at ART initiation, years old				
**≥50** **<50**	2·20 (0·52-9·34) 1·0 (Ref.)	0·282	3·07 (0·35-26·98) 1.0 (Ref.)	0·312
Comorbidities				
**Yes** **No**	1·50 (0·39-5·77) 1·0 (Ref.)	0·553	–	–
CDC stage C at HIV diagnosis				
**Yes** **No**	**13·01 (1·40-120·48)** 1·0 (Ref.)	**0·024**	1·90 (0·20-18·25) 1·0 (Ref.)	0·576
CD4+ T-cell <350/mm^3^ at ART initiation				
**Yes** **No**	1·67 (0·51-5·45) 1·0 (Ref.)	0·390	1·01 (0·26-3·88) 1·0 (Ref.)	0·984

IP-10, Interferon-inducible protein-10; MIG, Monokine induced by interferon-gamma; ART, antiretroviral treatment; OR, odds ratio; CI, confidence interval. Statistically significant values of variables are highlighted in bold.

Interestingly, the median CD4+ T count was <350 cells/mm3 regardless if individuals were aged >50 or <50 years (192 vs. 347 cells/mm^3^, p=0·145). Although, the median time from HIV diagnosis until ART initiation was higher in individuals who were aged ≥50 years than in individuals with ages <50 years, this difference was not statistically significant (66 vs. 44 days, respectively, p= 0.446).PWH with CDC stage C had higher baseline IP-10 levels (p=0·001) and MIG levels (p=0·024) than those with a CDC stage A. After ART initiation, both cytokines decreased, and no significant differences were observed among PWH with a CDC stage C vs. A after month 6 for MIG. However, IP-10 levels were higher in PWH with CDC stage C vs. A at month 12 (p=0·021) (**Figure 5B**). Indeed, a CDC stage C at ART initiation was identified as the only independent variable associated with having high levels of IP-10 at month 12 (aOR 13·01;95%CI:1·40-120·48, p=0·024), but not with high levels of MIG at month 12, in the multivariate analysis (**Table 2**).

PWH with CD4+ T count <350 cells/mm^3^ at ART initiation had higher IP-10 (p=0·002) and MIG levels (p=0·069, borderline significance) than those with CD4+ T count ≥350 cells/mm^3^. Subsequently, both cytokines decreased and matched those with CD4+ count ≥350 cells/mm^3^ at month 6 (**Figure 5C**). CD4+ T count <350 cells/mm^3^ neither was associated with high levels of IP-10 or MIG at month 12, in the multivariate analysis (**Table 2**).

### Impact of low-level viremia, TD or TND below 20 copies/mL on IP-10 and MIG levels, at month 12 after ART initiation

There were no differences in IP-10 and MIG plasma levels among individuals with TD or TND HIV-1 RNA below 20 copies/mL, or those with low-level viremia (HIV-1 RNA 20-199 copies/mL) at month 12 after ART initiation (**Table S2**).

### Impact of ARV regimen on IP-10 and MIG plasma levels

**Figure 6:**
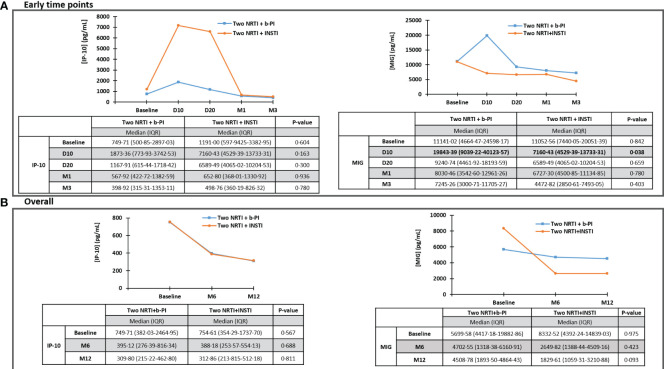
Impact of ARV regimen on IP-10 and MIG, throughout early time points **(A)** and overall (throughout 12 months) **(B)**. IP-10, Interferon-inducible protein-10; MIG, Monokine induced by interferon-gamma; ARV, antiretroviral; D10, day 10; D20, day 20; M1, month 1; M3, month 3; M6, month 6; M12, month 12; NRTI, nucleos(t)ide reverse transcriptase inhibitors; b-PI, boosted-protease inhibitor; INSTI, integrase strand transfer inhibitor. Statistically significant values of variables are highlighted in bold. IP-10 and MIG plasma levels are represented as plasma concentrations median values.

At day 10 after ART initiation, we found MIG levels significantly lower (in contrast with IP-10) in individuals who initiated two NRTIs plus INSTI vs. two NRTIs plus b-PI (p=0·038) (**Figure 6A**). No significant impact on IP-10 and MIG plasma levels was identified over time after day 10, regardless of the initial ART (**Figure 6B**).

## Discussion

In this prospective cohort analysis, IP-10 and MIG plasma levels showed an early, significant, and consistent decline just a few days after ART initiation and throughout 48 months.

There was significant global parallelism of plasma IP-10 and MIG decreases with HIV-1-RNA suppression and CD4+ T cell count recovery.

These findings are concordant with those previously reported in a French cohort of ART-naïve individuals, but where IP-10 and MIG plasma levels were measured only at baseline and 24 months after ART initiation (15). We were able, in addition, to show a significant reduction of these biomarkers at very early time points (20 days, specifically for IP-10), therefore recognizing both IP-10 and MIG as early ART response surrogate biomarkers. This strongly suggests that these biomarkers are impacted mainly by unsuppressed HIV-1 viremia and they decrease rapidly paralleling the significant HIV-1 RNA decay seen in this cohort just 1 month after ART initiation. The CD4 + T cell count recovery took a longer time, approximately 6 months.

At baseline, we found significantly higher IP-10 and MIG levels in PWH aged ≥ 50 years. A French cohort (n=139) reported similar baseline levels of IP-10 and MIG among age strata, but groups were defined as those aged ≤35 years, those with ages ranging from 35 to 44 years, and those aged ≥44 years (15). Of note, the number of individuals aged ≥50 years included in the upper stratum (>44 years) is unknown. Thus, the inclusion of individuals with ages ranging from 44 to 49 years in the same group could have biased the overall analysis. In our study, after ART initiation, both cytokines showed a sharp reduction in PWH aged ≥50 years, and at month 12, IP-10 levels matched those of PWH aged <50 years. However, the MIG level decline was slower and differences were still significant at month 12. Older age was associated with higher levels of IP-10 at month 24 after ART initiation (OR 1·6 per 10 years older, p=0·047, in multivariate analysis) in the previous French cohort (15). In addition, in an Australian cohort, plasma IP-10 levels were more likely to remain higher in older individuals with comorbidities (frailty, hypertension, and coronary heart disease), which correlates with other traditional cardiovascular disease surrogate plasma biomarkers, including C-reactive protein, IL-6, and fibrinogen (21). Importantly, older age ranges were not defined in either of these cohorts. Although these data could suggest that persistent increases in IP-10 would not be impacted by age by itself, but by existing comorbidities, in our multivariate analysis, older age (≥50 years) and comorbidities were not associated with persistent high levels of IP-10 at month 12 after ART initiation, and neither was it associated with high levels of MIG. The higher rates of HIV-1 RNA suppression achieved with current ART regimens could therefore overcome baseline differences seen in previous cohorts.

In our study, PWH with CDC stage C had higher baseline IP-10 and MIG levels than those with CDC stage A. According to our data, IP-10 increases have been described in PWH with opportunistic infections (tuberculosis and cryptosporidiosis) (7). Baseline levels of IP-10 (but not MIG) were significantly higher in individuals with AIDS-defining events in the aforementioned French cohort, and this condition was not associated with subsequent persistently elevated levels of both cytokines (non-adjusted analysis) at month 24 after ART initiation (15). In our cohort, we were able to find a sharp decrease in both cytokines upon ART initiation in PWH with baseline CDC stage C, and no significant differences were subsequently observed between CDC stages C vs. A, at month 6 after ART initiation. However, CDC stage C was identified as an independent variable associated with a risk of having high levels of IP-10 at month 12 after ART initiation, in the adjusted multivariate analysis.

Interestingly, we observed that PWH with CD4+ count <350 cells/mm^3^ at ART initiation had higher baseline IP-10 and MIG levels than those with CD4+ count ≥350 cells/mm^3^. The difference was significant for IP-10 and borderline significant for MIG. Upon ART initiation, both cytokine levels decreased and matched those of PWH with CD4+ count ≥350 cells/mm^3^ at month 6 and thereafter. A Brazilian cohort found that individuals with CD4+ count <350 cells/mm^3^ at ART initiation showed higher IP-10 levels compared to those with CD4+ count >350 cells/mm^3^. In that cohort, individuals who initiated ART with CD4+ count >350 cells/mm^3^ achieved normalization of plasma IP-10 to levels similar to those of healthy individuals after undergoing ART for a median of 12 months. However, the evolution of IP-10 at 12 months was not compared between PWH with CD4+ count < or > 350 cells/mm^3^ at ART initiation (22).

In our study, there were no differences in IP-10 and MIG plasma levels among individuals with TND or TD HIV-1 RNA below 20 copies/mL or those with low-level viremia (20-199 copies/mL) at month 12 after ART initiation. This is in agreement with data retrieved from a Swedish case-control study, where IP-10 did not show significant differences between individuals with detected viremia (50-999 copies/mL) and those with persistent virological suppression (HIV-1 RNA <50 copies/mL) after 12 months following ART initiation (23). However, we had limited power in the number of individuals with these characteristics, which precludes a stronger conclusion regarding this association.

Interestingly, on day 10 after ART initiation, MIG (but not IP-10) levels showed a significant decrease in individuals who initiated an INSTI-based regimen (vs. a b-PI-based one). This could be influenced by the well-documented significantly faster initial HIV-1 RNA decay kinetics of INSTIs (24, 25). Importantly, this suggests that MIG is more impacted than IP-10 by this initial plasma HIV-1 RNA decay under INSTIs, and more influenced by that than by the CD4+ T cell count recovery or homeostasis. Even though the differences were only significant at day 10, we saw slower decreases in MIG values with b-PI, which remained numerically higher than those in INSTI-treated individuals up to 12 months. In a French cohort, the decline in IP-10 and MIG levels was significantly smaller with ritonavir-boosted atazanavir than with efavirenz, while no significant difference was found between ritonavir-boosted lopinavir and efavirenz at 24 months after initiating ART (26). However, data were lacking regarding currently recommended INSTI as a third drug. In this regard, in an Italian cohort that divided the individuals into groups according to the third drug at the time of enrollment, the decline in IP-10 plasma levels was significantly lower among individuals receiving non-nucleoside reverse transcriptase inhibitors compared to the b-PI and INSTI group after at least 12 months following ART initiation (27).

Despite both IP-10 and MIG being pro-inflammatory biomarkers, the subtle differences found in their plasma dynamics in the present study could suggest potentially diverse pathophysiological underlying mechanisms. In contrast to MIG, IP-10 (known as being impacted as prematurely as 6 days after primary HIV-1 infection) showed significant declines as early as 20 days after ART initiation. Intriguingly, IP-10 levels remained high at month 12 in PWH with CDC-stage C at HIV diagnosis. A higher reservoir size (partly mediated by IP-10) (6, 28) could contribute to these high IP-10 levels in this subset of individuals. In this regard, it has been postulated that IP-10 could be produced as part of a second wave of cytokine responses, following toll-like receptor (TLR)7/8 stimulation and activation of antigen-presenting cells, though not to the same extent as following primary virus stimulation (12).

Altogether, these results suggest PWH who start ART on time (CD4+ T cell count ≥350 cells/mm^3^) and achieve levels of HIV-1 viremia below 200 copies/mL after undergoing ART for 12 months, have limited levels of chronic inflammation to impact MIG and IP-10 12 months after ART initiation. Nearly half of PWH in this cohort had CD4+ counts <350 cells/mm3 at baseline. Initiating ART during the first year of HIV infection reduces numerous biomarkers of inflammation and immune activation that are impacted by different inflammatory pathways. This response is more remarkable during acute HIV infection, potentially because of an early effect on attaining a smaller HIV-1 latent reservoir (partly mediated through IP-10) (6) and viral diversity (28). A baseline CDC-stage C is associated with a greater HIV reservoir and higher levels of persistent inflammation, with high IP-10 levels at month 12 after ART initiation in our study. These data suggest that IP-10 would be more impacted than MIG by a greater HIV reservoir. In turn, MIG seems to be more impacted than IP-10 by initial plasma HIV-1 RNA decay, as evidenced by a greater decline with INSTI-based regimens (vs. a b-PI-based one).

Overall, these findings reinforce the recommendation of early ART, prioritizing INSTI-based regimens, resulting in a decrease in chronic immune activation and inflammation.

At baseline, PWH aged >50 years, a CDC-stage C, or CD4+ T count <350 cells/mm^3^ had higher levels of both IP-10 and MIG levels. All these variables are associated with late HIV diagnosis. This finding reinforces the need to achieve timely diagnosis of individuals with occult or unknown HIV infection. This study adds information regarding early and late time points in the dynamics of IP-10 and MIG plasma levels, as well as in current scenarios in clinical practice: PWH aged ≥50 years, INSTI-based ART, and low-level and TND viremia.

Further analyses are required on the potential impact on plasma IP-10 and MIG and subsequent chronic immune dysfunction of new ART regimens, including dolutegravir-based two-drug regimens and long-acting formulations, as well as in people with persistent immune discordance.

Our study has limitations. The small sample size could underpower some of the analyses, especially those evaluating particular scenarios. Despite internal quality control and the prospective design, in a real-life cohort study, errors and heterogeneity in data gathering and recording cannot be ruled out. Finally, IP-10 and MIG could be impacted by other simultaneous uncontrolled conditions, such as viral or bacterial coinfections, which could act as potential confounding factors.

In conclusion, in this prospective cohort, IP-10 and MIG arise as soluble plasma biomarkers that showed an early, significant, and consistent decline in their plasma levels just a few days after ART initiation, paralleling the HIV-1 RNA decay and CD4+ T cell recovery. PWH with ages ≥50 years, CDC stage C, or PWH with CD4+ count <350 cells/mm^3^ had higher baseline IP-10 and MIG levels, with sharp subsequent declines. All of them matched their counterparts several months following ART initiation. PWH treated with INSTI-based regimens had faster declines of MIG as early as day 10. Overall, these findings suggest that both IP-10 and MIG are sensitive biomarkers of early antiretroviral treatment response, and are mainly impacted by plasma HIV-1 viremia, low CD4+ counts, and late ART initiation. Starting ART with CD4+ T cell count >350 cells/mm^3^ had limited impact on MIG or IP-10 levels. Once ART has been initiated, having TD HIV-1 RNA below 20 copies/mL or even low-level viremia (<200 copies/mL) did not impact MIG or IP-10 levels. These issues merit further investigation in larger cohorts and evaluation of new antiretroviral treatment strategies.

## Data availability statement

The original contributions presented in the study are included in the article/**Supplementary Material**. Further inquiries can be directed to the corresponding author.

## Ethics statement

The study was approved by the regional Ethic Committee (CAEIG), an external scientific and ethics committee of the HIV Biobank (Spanish HIV/AIDS Research Network).The studies were conducted in accordance with the local legislation and institutional requirements. The participants provided their written informed consent to participate in this study.

## Author contributions

HÁ: Conceptualization, Writing – original draft, Writing – review & editing. AG-V: Writing – review & editing. AM: Writing – review & editing. AS-A: Writing – review & editing. BC-C: Methodology, Writing – review & editing. AP-G: Writing – review & editing. JA-D: Methodology, Writing – review & editing. IM-B: Methodology, Writing – review & editing. MG-R: Writing – review & editing. SM: Writing – review & editing. TA: Writing – review & editing. MM-A: Writing – review & editing. EB: Writing – review & editing. CG: Writing – review & editing. JML: Conceptualization, Writing – original draft, Writing – review & editing. EP: Conceptualization, Writing – original draft, Writing – review & editing.

## Centers and investigators involved in CoRIS

Please see the **Supplementary file Data Sheet 2** for the full list of members.

## Supplementary material

Supplementary Figure S1 Dynamic changes in IP-10 plasma levels and CD4+ T-cell **(A)**; dynamic changes in IP-10 plasma levels and HIV-1 RNA **(B)**; dynamic changes in MIG plasma levels and CD4+ T-cell **(C)**; dynamic changes in MIG plasma levels and HIV-1 RNA **(D)**, throughout 12 months after ART initiation. IP-10, Interferon-inducible protein 10; MIG, Monokine induced by interferon-gamma; ART, antiretroviral treatment; M6, month 6; M12, month 12. Global tendency (throughout all time points represented in graph) p value, calculated with the Friedman Test. IP-10, MIG, CD4+ T-cell count and HIV-1 RNA plasma levels are represented as plasma concentrations median values.

Supplementary Figure S2 Correlation between IP-10 plasma levels and CD4+ T-cell count **(A)** and correlation between IP-10 plasma levels and HIV-1 RNA **(B)**, throughout 12 months after ART initiation. IP-10, Interferon-inducible protein-10; ART, antiretroviral treatment; r, correlation coefficient Spearman. Statistically significant values of variables are highlighted in bold.

Supplementary Figure S3 Correlation between MIG plasma levels and CD4+ T-cell count **(A)** and correlation between MIG plasma levels and HIV-1 RNA **(B)**, throughout 12 months after ART initiation. MIG, Monokine induced by interferon-gamma; ART, antiretroviral treatment; r, correlation coefficient Spearman. Statistically significant values of variables are highlighted in bold.

Supplementary Figure S4 Correlation between IP-10 plasma levels and CD4+ T-cell count **(A)** and correlation between IP-10 plasma levels and HIV-1 RNA **(B)**, throughout early time points after ART initiation. IP-10, Interferon-inducible protein-10; ART, antiretroviral treatment; r, correlation coefficient Spearman. Statistically significant values of variables are highlighted in bold.

Supplementary Figure S5 Correlation between MIG plasma levels and CD4+ T-cell count **(A)** and correlation between MIG plasma levels and HIV-1 RNA **(B)**, throughout early time points after ART initiation. MIG, Monokine induced by interferon-gamma; ART, antiretroviral treatment; r, correlation coefficient Spearman. Statistically significant values of variables are highlighted in bold.

Supplementary Table S1 Dynamic changes in CD4+ T-cell count and HIV-1 RNA after ART initiation overall (throughout 12 months) **(A)**. Dynamic changes in CD4+ T-cell count and HIV-1 RNA after ART initiation throughout early time points **(B)**. ART, antiretroviral treatment; D10, day 10; D20, day 20; M1, month 1; M3, month 3; M6, month 6; M12, month 12; IQR, interquartile range. Statistically significant values of variables are highlighted in bold.

Supplementary Table S2 Impact of low-level viremia, target detected or target not detected below 20 copies/mL on IP-10 and MIG plasma levels, 12 months after ART initiation. IP-10, Interferon-inducible protein-10; MIG, Monokine induced by interferon-gamma; M12, month 12; n, number of individuals. Statistically significant values of variables are highlighted in bold. * Low-level viremia is defined as HIV-1 RNA 20-199copies/mL.

Supplementary Data Sheet 2 Centres and investigators involved in CoRIS.
