# Supplementary material for: IP-10 and MIG are sensitive markers of early virological response to HIV-1 integrase inhibitors
Source: Front Immunol. 2023 Oct 18;14:1257725. doi: 10.3389/fimmu.2023.1257725 (PMC10619723; doi:10.3389/fimmu.2023.1257725)
Supplement: Supplementary Figure S1 — Dynamic changes in IP-10 plasma levels and CD4+ T-cell (A); dynamic changes in IP-10 plasma levels and HIV-1 RNA (B); dynamic changes in MIG plasma levels and CD4+ T-cell (C); dynamic changes in MIG plasma levels and HIV-1 RNA (D), throughout 12 months after ART initiation. IP-10, Interferon-inducible protein 10; MIG, Monokine induced by interferon-gamma; ART, antiretroviral treatment; M6, month 6; M12, month 12. Global tendency (throughout all time points represented in graph) p value, calculated with the Friedman Test. IP-10, MIG, CD4+ T-cell count and HIV-1 RNA plasma levels are represented as plasma concentrations median values. [file DataSheet_1.zip › TableS1.pdf]

A. Overall

	CD4+ T-cells/mm3	
	Median (IQR)	**P-value** (vs. baseline)
**Baseline**	407·28 (149·00-671·75)	-
**M6**	581·00 (320·25-839·50)	**<0·001**
**M12**	564·50 (386·75-868·50)	**<0·001**

	HIV-1 RNA (log10 copies/mL)	
	Median (IQR)	**P-value** (vs. baseline)
**Baseline**	4·76 (4·15-5·53)	-
**M6**	1·69 (1·51-2·19)	**<0·001**
**M12**	1·64 (1·39-1·92)	**0·002**

B. Early time points

	CD4+ T-cells/mm^3	
	Median	P-value (vs. baseline)
**Baseline**	323·00 (54·50-663·50)	-
**D10**	-	-
**D20**	-	-
**M1**	451·00 (198·50-653·00)	1·000
**M3**	515·00 (249·75-734·25)	0·141

	HIV-1 RNA (log_{10} copies/mL)	
	Median	P-value (vs. baseline)
**Baseline**	4·94 (4·29-5·74)	-
**D10**	-	-
**D20**	-	-
**M1**	2·15 (1·91-2·85)	**<0·001**
**M3**	1·84 (1·61-2·51)	**<0·001**
