# Supplementary material for: IP-10 and MIG are sensitive markers of early virological response to HIV-1 integrase inhibitors
Source: Front Immunol. 2023 Oct 18;14:1257725. doi: 10.3389/fimmu.2023.1257725 (PMC10619723; doi:10.3389/fimmu.2023.1257725)
Supplement: Supplementary Figure S1 — Dynamic changes in IP-10 plasma levels and CD4+ T-cell (A); dynamic changes in IP-10 plasma levels and HIV-1 RNA (B); dynamic changes in MIG plasma levels and CD4+ T-cell (C); dynamic changes in MIG plasma levels and HIV-1 RNA (D), throughout 12 months after ART initiation. IP-10, Interferon-inducible protein 10; MIG, Monokine induced by interferon-gamma; ART, antiretroviral treatment; M6, month 6; M12, month 12. Global tendency (throughout all time points represented in graph) p value, calculated with the Friedman Test. IP-10, MIG, CD4+ T-cell count and HIV-1 RNA plasma levels are represented as plasma concentrations median values. [file DataSheet_1.zip › TableS2.pdf]

	Low-level viremia* or target detected HIV-1 RNA but <20 copies/mL n=25	Target not detected HIV-1 RNA n=13	P-value
**IP-10 (M12), pg/mL**	406·11 (303·52-724·81)	500·19 (322·29-628·97)	0·540
**MIG (M12), pg/mL**	3757·33 (1844·87-5299·09)	2482·43 (1911·67-3793·48)	0·296

	Target not detected or detected HIV-1 RNA but <20 copies/mL n=24	Low-level viremia* n=14	P-value
**IP-10 (M12), pg/mL**	454·57 (309·47-607·01)	440·13 (340·21-884·08)	0.564
**MIG (M12), pg/mL**	2956·83 (2099·21-4502·49)	3757·33 (1585·11-5821·31)	0·829
