# Supplementary figures and images for: IP-10 and MIG are sensitive markers of early virological response to HIV-1 integrase inhibitors
Source: Front Immunol. 2023 Oct 18;14:1257725. doi: 10.3389/fimmu.2023.1257725 (PMC10619723; doi:10.3389/fimmu.2023.1257725)

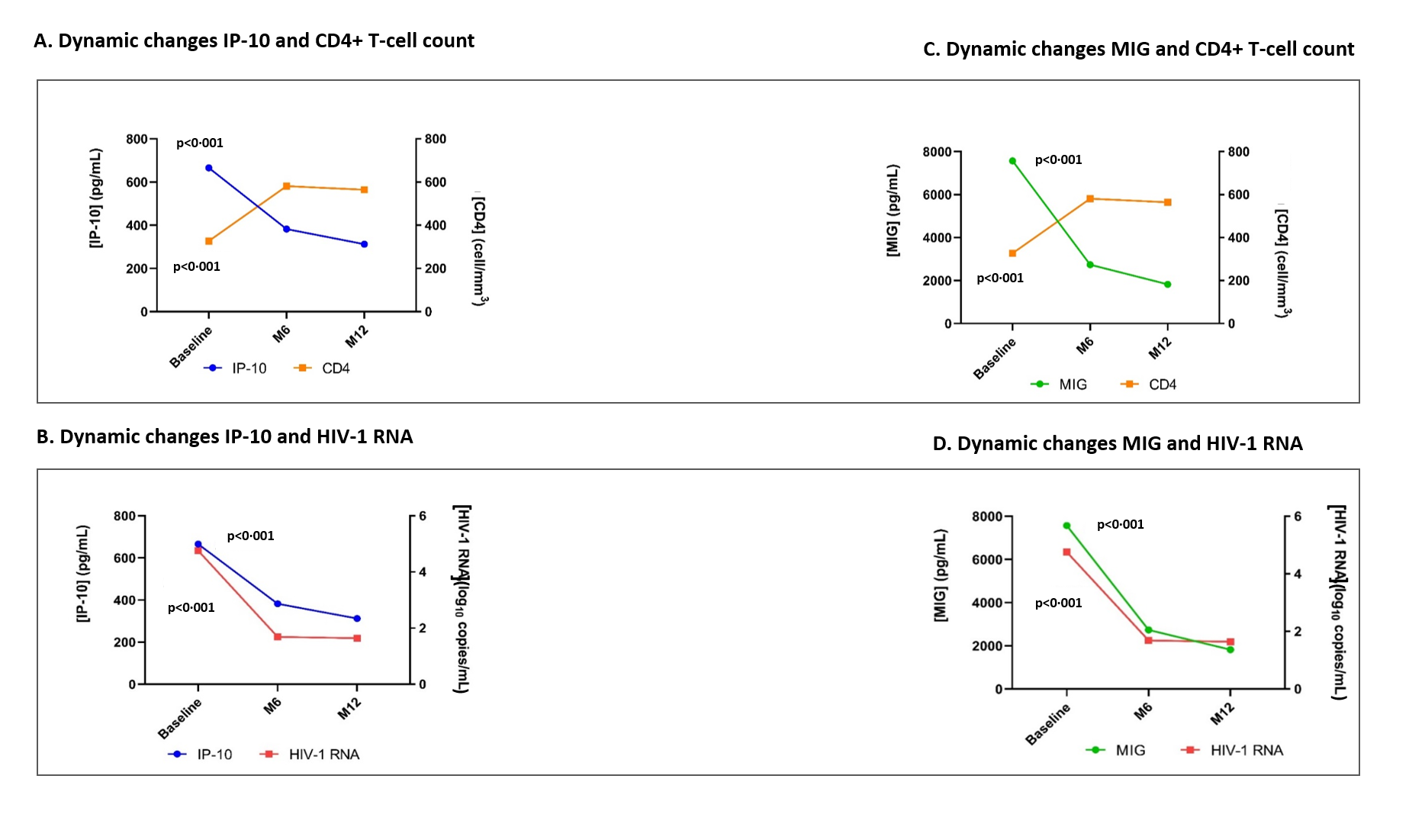

Supplement: Supplementary Figure S1 — Dynamic changes in IP-10 plasma levels and CD4+ T-cell (A); dynamic changes in IP-10 plasma levels and HIV-1 RNA (B); dynamic changes in MIG plasma levels and CD4+ T-cell (C); dynamic changes in MIG plasma levels and HIV-1 RNA (D), throughout 12 months after ART initiation. IP-10, Interferon-inducible protein 10; MIG, Monokine induced by interferon-gamma; ART, antiretroviral treatment; M6, month 6; M12, month 12. Global tendency (throughout all time points represented in graph) p value, calculated with the Friedman Test. IP-10, MIG, CD4+ T-cell count and HIV-1 RNA plasma levels are represented as plasma concentrations median values. [file DataSheet_1.zip › Figure S1.tif]

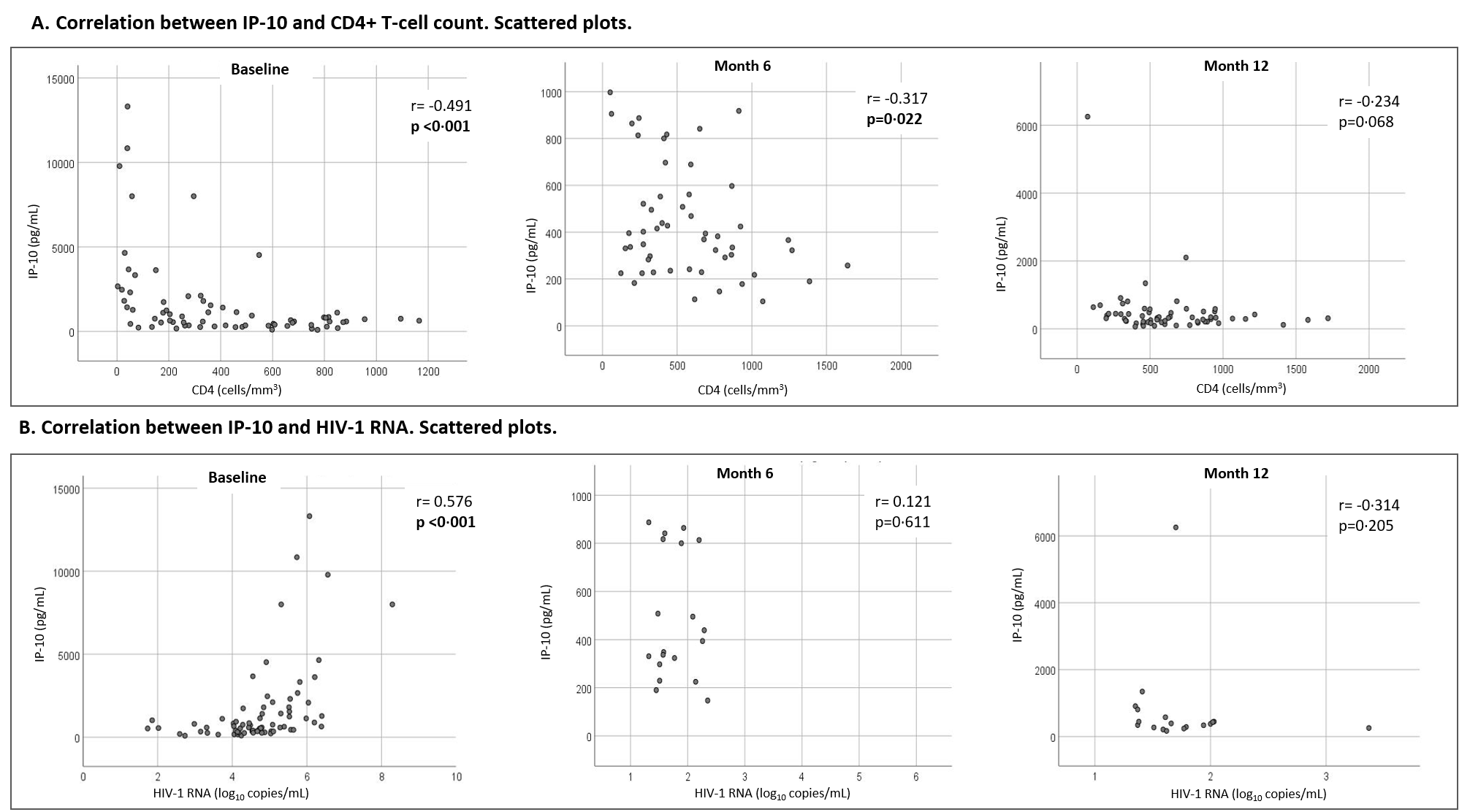

Supplement: Supplementary Figure S1 — Dynamic changes in IP-10 plasma levels and CD4+ T-cell (A); dynamic changes in IP-10 plasma levels and HIV-1 RNA (B); dynamic changes in MIG plasma levels and CD4+ T-cell (C); dynamic changes in MIG plasma levels and HIV-1 RNA (D), throughout 12 months after ART initiation. IP-10, Interferon-inducible protein 10; MIG, Monokine induced by interferon-gamma; ART, antiretroviral treatment; M6, month 6; M12, month 12. Global tendency (throughout all time points represented in graph) p value, calculated with the Friedman Test. IP-10, MIG, CD4+ T-cell count and HIV-1 RNA plasma levels are represented as plasma concentrations median values. [file DataSheet_1.zip › Figure S2.tif]

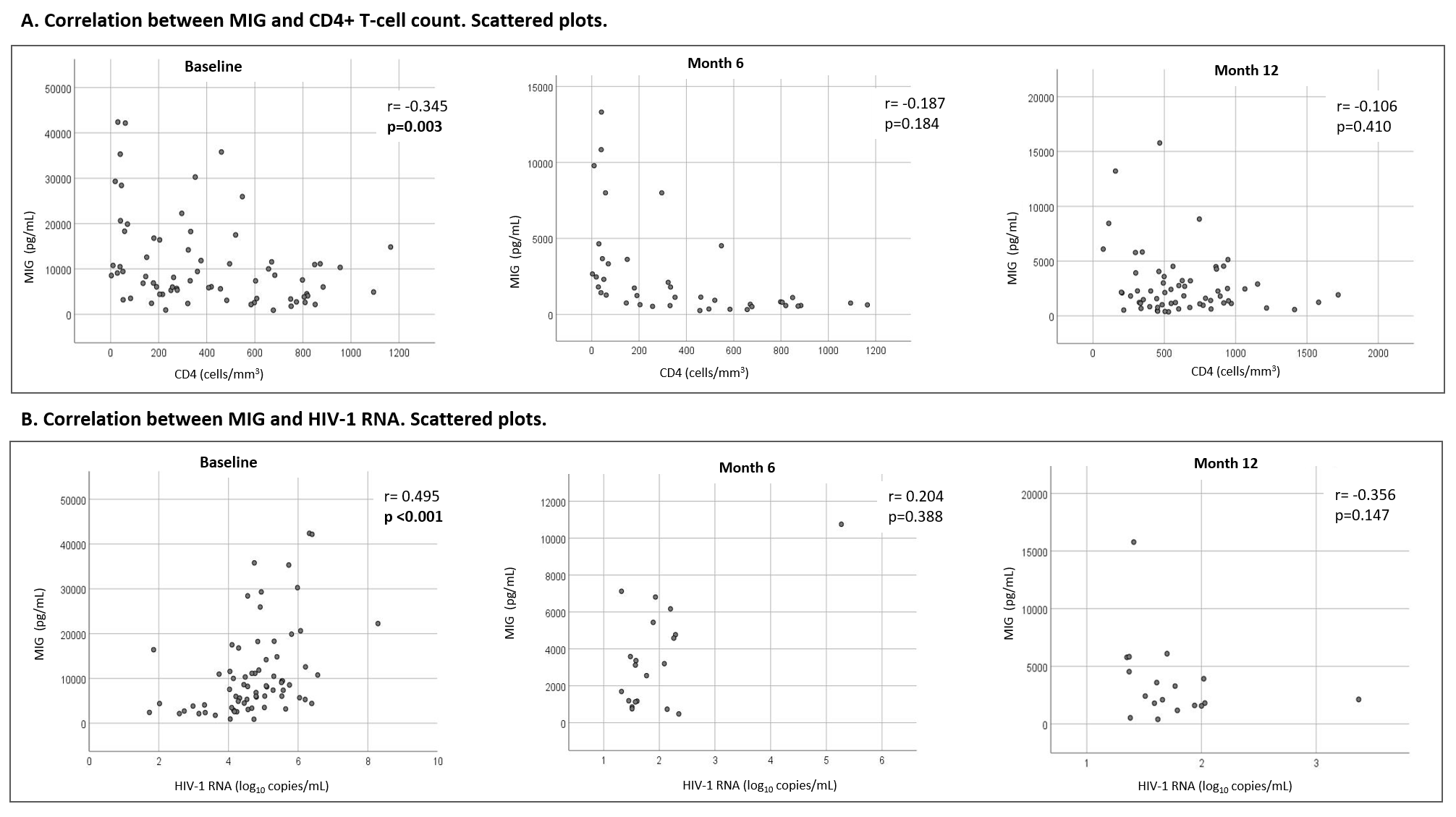

Supplement: Supplementary Figure S1 — Dynamic changes in IP-10 plasma levels and CD4+ T-cell (A); dynamic changes in IP-10 plasma levels and HIV-1 RNA (B); dynamic changes in MIG plasma levels and CD4+ T-cell (C); dynamic changes in MIG plasma levels and HIV-1 RNA (D), throughout 12 months after ART initiation. IP-10, Interferon-inducible protein 10; MIG, Monokine induced by interferon-gamma; ART, antiretroviral treatment; M6, month 6; M12, month 12. Global tendency (throughout all time points represented in graph) p value, calculated with the Friedman Test. IP-10, MIG, CD4+ T-cell count and HIV-1 RNA plasma levels are represented as plasma concentrations median values. [file DataSheet_1.zip › Figure S3.tif]

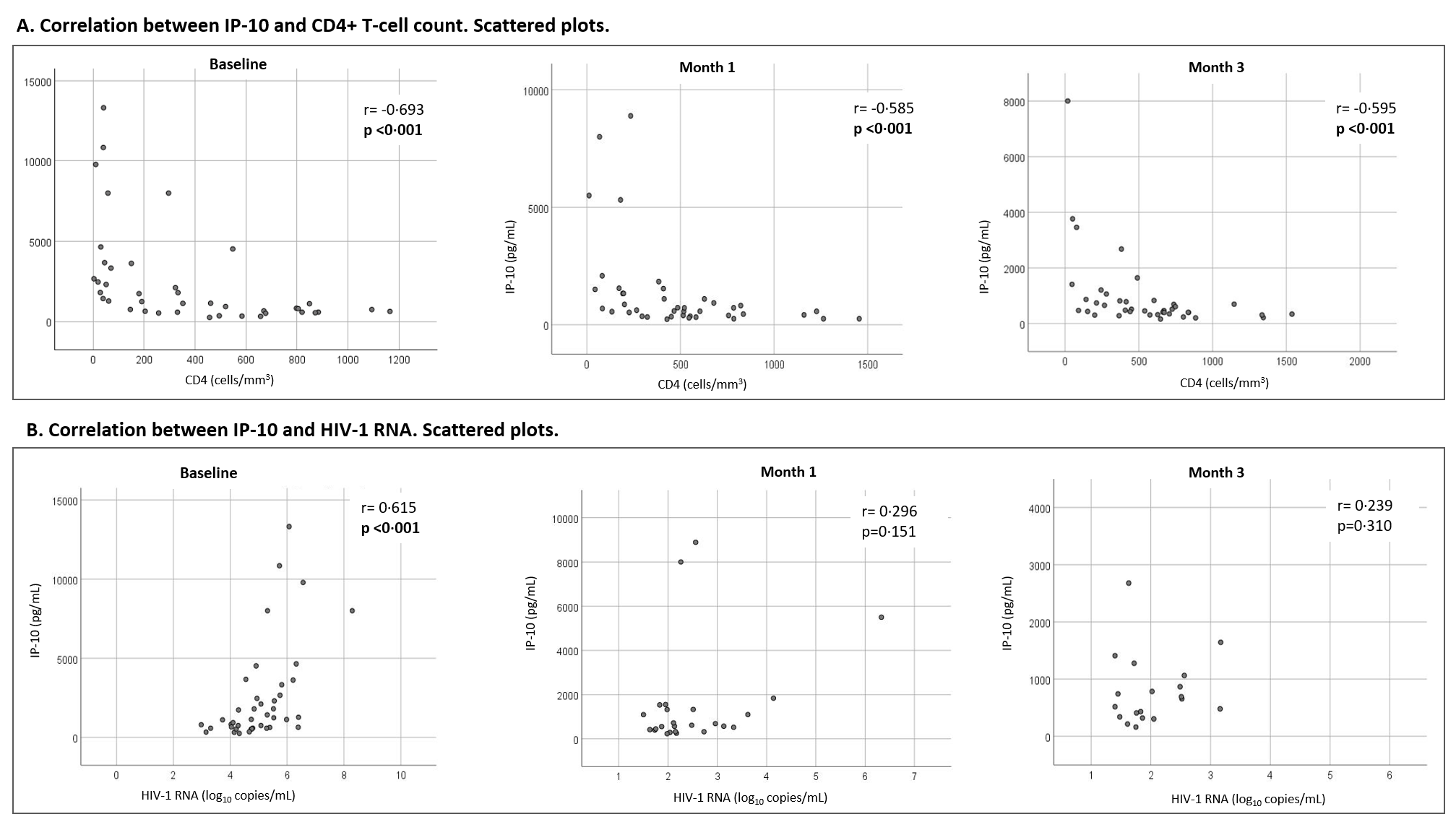

Supplement: Supplementary Figure S1 — Dynamic changes in IP-10 plasma levels and CD4+ T-cell (A); dynamic changes in IP-10 plasma levels and HIV-1 RNA (B); dynamic changes in MIG plasma levels and CD4+ T-cell (C); dynamic changes in MIG plasma levels and HIV-1 RNA (D), throughout 12 months after ART initiation. IP-10, Interferon-inducible protein 10; MIG, Monokine induced by interferon-gamma; ART, antiretroviral treatment; M6, month 6; M12, month 12. Global tendency (throughout all time points represented in graph) p value, calculated with the Friedman Test. IP-10, MIG, CD4+ T-cell count and HIV-1 RNA plasma levels are represented as plasma concentrations median values. [file DataSheet_1.zip › Figure S4.tif]

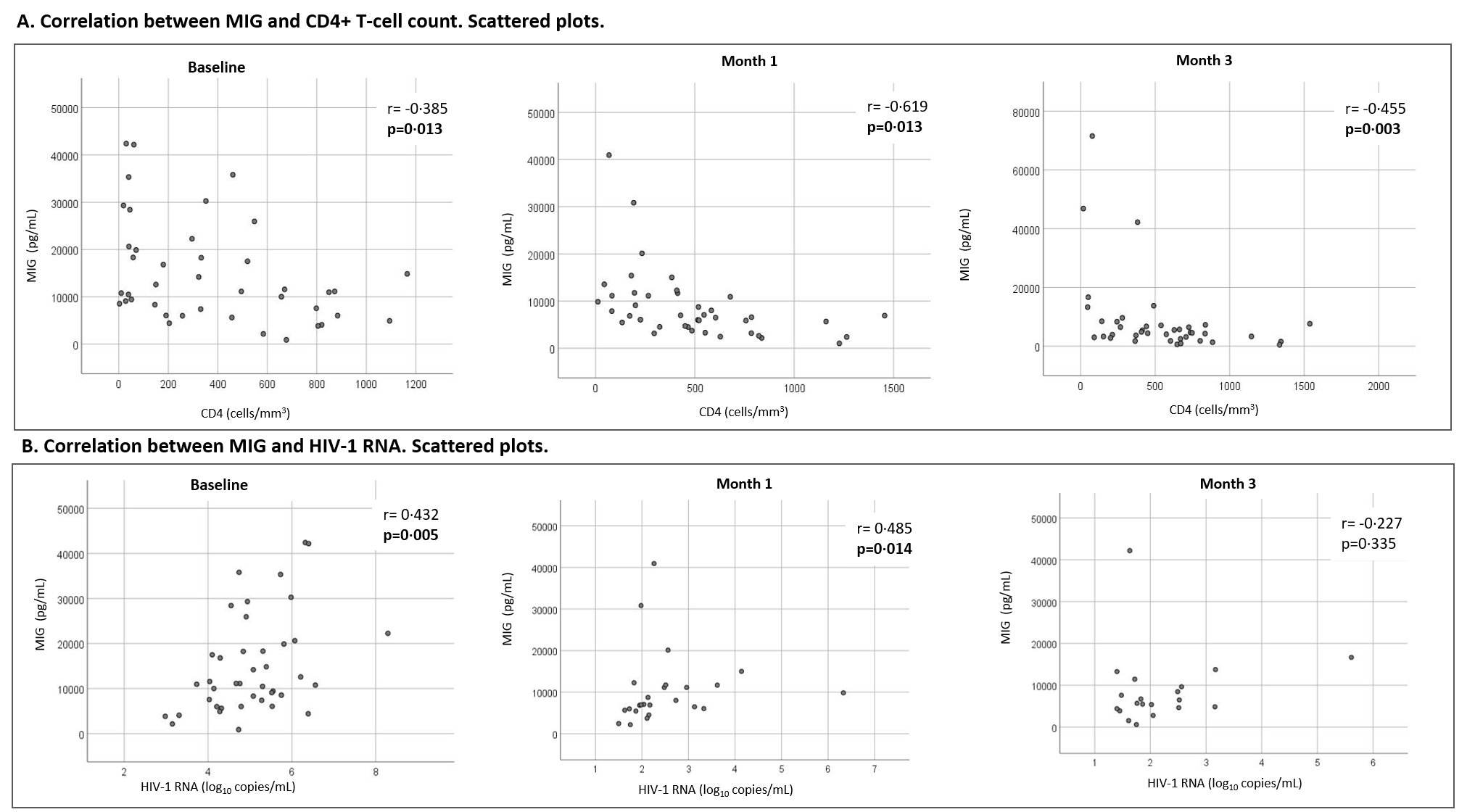

Supplement: Supplementary Figure S1 — Dynamic changes in IP-10 plasma levels and CD4+ T-cell (A); dynamic changes in IP-10 plasma levels and HIV-1 RNA (B); dynamic changes in MIG plasma levels and CD4+ T-cell (C); dynamic changes in MIG plasma levels and HIV-1 RNA (D), throughout 12 months after ART initiation. IP-10, Interferon-inducible protein 10; MIG, Monokine induced by interferon-gamma; ART, antiretroviral treatment; M6, month 6; M12, month 12. Global tendency (throughout all time points represented in graph) p value, calculated with the Friedman Test. IP-10, MIG, CD4+ T-cell count and HIV-1 RNA plasma levels are represented as plasma concentrations median values. [file DataSheet_1.zip › Figure S5.tif]
