# Supplementary material for: IP-10 and MIG are sensitive markers of early virological response to HIV-1 integrase inhibitors
Source: Front Immunol. 2023 Oct 18;14:1257725. doi: 10.3389/fimmu.2023.1257725 (PMC10619723; doi:10.3389/fimmu.2023.1257725)
Supplement: Supplementary Data Sheet 2 — Centres and investigators involved in CoRIS. [file DataSheet_2.docx]

**CENTRES AND INVESTIGATORS INVOLVED IN CoRIS**

**CoRIS Executive Committee:**

Santiago Moreno, Inma Jarrín, David Dalmau, M Luisa Navarro, M Isabel González, Federico Garcia, Eva Poveda, Jose Antonio Iribarren, Félix Gutiérrez, Rafael Rubio, Francesc Vidal, Juan Berenguer, Juan González, M Ángeles Muñoz-Fernández.

**Centres and investigators involved in the CoRIS cohort are listed below:**

**CoRIS Coordination Unit**

Inmaculada Jarrín, Cristina Moreno, Marta Rava, Rebeca Izquierdo, Cristina Marco, Julián Puente, Teresa Gómez García.

**BioBanK HIV Hospital General Universitario Gregorio Marañón**

M Ángeles Muñoz-Fernández, Roxana Juárez.

**Hospital General Universitario de Alicante (Alicante)**

Joaquín Portilla, Irene Portilla, Esperanza Merino, Gema García, Iván Agea, José Sánchez-Payá, Juan Carlos Rodríguez, Livia Giner, Sergio Reus, Vicente Boix, Diego Torrus, Verónica Pérez, Julia Portilla, Héctor Pinargote.

**Hospital Universitario de Canarias (San Cristóbal de la Laguna)**

María Remedios Alemán, Ana López Lirola, Dácil García, Felicitas Díaz-Flores, M Mar Alonso, Ricardo Pelazas, María Inmaculada Hernández, Lucia Romero, Abraham Bethencourt, Daniel Rodríguez.

**Hospital Universitario Central de Asturias (Oviedo)**

Víctor Asensi, María Eugenia Rivas-Carmenado, Rebeca Cabo Magadan, Javier Díaz-Arias

**Hospital Universitario 12 de Octubre (Madrid)**

Federico Pulido, Rafael Rubio, Otilia Bisbal, M Asunción Hernando, David Rial, María de Lagarde, Adriana Pinto, Laura Bermejo, Mireia Santacreu, Roser Navarro, Juan Martín Torres.

**Servicio de Enfermedades Infecciosas. Hospital Universitario Donostia. Instituto de Investigación BioDonostia (Donostia-San Sebastián)**

José Antonio Iribarren, M José Aramburu, Xabier Camino, Miguel Ángel Goenaga, M Jesús Bustinduy, Harkaitz Azkune, Maialen Ibarguren, Xabier Kortajarena, Ignacio Álvarez-Rodriguez, Leire Gil, Francisco Carmona-Torre, Ana Bayona Carlos, Maialen Lekuona Sanz.

**Hospital General Universitario De Elche (Elche)**

Félix Gutiérrez, Catalina Robledano, Mar Masiá, Sergio Padilla, Araceli Adsuar, Rafael Pascual, Marta Fernández, Antonio Galiana, José Alberto García, Xavier Barber, Javier García Abellán, Guillermo Telenti, Lucía Guillén, Ángela Botella, Paula Mascarell, Mar Carvajal, Alba de la Rica, Carolina Ding, Lidia García-Sánchez, Nuria Ena, Leandro López, Jennifer Vallejo, Nieves Gonzalo-Jiménez, Montserrat Ruiz, Christian Ledesma, Santiago López, María Espinosa, Ana Quiles, María Andreo.

**Hospital Universitari Germans Trias i Pujol (Can Ruti) (Badalona)**

Roberto Muga, Arantza Sanvisens, Daniel Fuster.

**Hospital General Universitario Gregorio Marañón (Madrid)**

Juan Carlos López Bernaldo de Quirós, Isabel Gutiérrez, Juan Berenguer, Margarita Ramírez, Paloma Gijón, Teresa Aldamiz-Echevarría, Francisco Tejerina, Cristina Diez, Leire Pérez, Chiara Fanciulli, Saray Corral.

**Hospital Universitari de Tarragona Joan XXIII (Tarragona)**

Joaquín Peraire, Anna Rull, Anna Martí, Consuelo Viladés, Beatriz Villar, Lluïsa Guillem, Montserrat Olona, Graciano García-Pardo, Frederic Gómez-Bertomeu, Verónica Alba, Silvia Chafino, Alba Sánchez.

**Hospital Universitario y Politécnico de La Fe (Valencia)**

Marta Montero, María Tasias, Eva Calabuig, Miguel Salavert, Juan Fernández, Rosa Blanes.

**Hospital Universitario La Paz/IdiPAZ (Madrid)**

Juan González-García, Ana Delgado-Hierro, José Ramón Arribas, Víctor Arribas, José Ignacio Bernardino, Carmen Busca, Joanna Cano, Julen Cardiñanos, Juan Miguel Castro, Luis Escosa, Iker Falces, Pedro Herranz, Víctor Hontañón, Milagros García, Alicia González-Baeza, M Luz Martín-Carbonero, Mario Mayoral, Mª Jose Mellado, Rafael Micán, Rosa de Miguel, Rocío Montejano, Mª Luisa Montes, Victoria Moreno, Luis Ramos~~,~~ Berta Rodés, Talía Sainz, Elena Sendagorta, Eulalia Valencia.

**Hospital San Pedro Centro de Investigación Biomédica de La Rioja (CIBIR) (Logroño)**

José Ramón Blanco, Laura Pérez-Martínez, José Antonio Oteo, Valvanera Ibarra, Luis Metola, Mercedes Sanz.

**Hospital Universitario Miguel Servet (Zaragoza)**

Rosa Martínez, Desiré Gil, Álvaro Cecilio, Ruth Caballero, María Aranzazu Caudevilla.

**Hospital Universitari Mutua Terrassa (Terrassa)**

David Dalmau, Marina Martinez, Angels Jaén, Mireia Cairó, Javier Martinez-Lacasa, Roser Font, Laura Gisbert.

**Hospital Universitario de Navarra (Pamplona)**

María Rivero, Maider Goikoetxea, María Gracia, Carlos Ibero, Estela Moreno, Jesús Repáraz, Fernando Baigorria.

**Parc Taulí Hospital Universitari (Sabadell)**

Gemma Navarro, Manel Cervantes Garcia, Sonia Calzado Isbert, Marta Navarro Vilasaro.

**Hospital Universitario de La Princesa (Madrid)**

Ignacio de los Santos, Alejandro de los Santos, Lucio García-Fraile, Enrique Martín, Ildefonso Sánchez-Cerrillo, Marta Calvet, Ana Barrios, Azucena Bautista, Carmen Sáez, Marianela Ciudad, Ángela Gutiérrez, María Aguilera García.

**Hospital Universitario Ramón y Cajal (Madrid)**

Santiago Moreno, Santos del Campo, José Luis Casado, Fernando Dronda, Ana Moreno, M Jesús Pérez, Sergio Serrano, Mª Jesús Vivancos, Javier Martínez-Sanz, Alejandro Vallejo, Matilde Sánchez, José Antonio Pérez-Molina, José Manuel Hermida.

**Hospital General Universitario Reina Sofía (Murcia)**

Enrique Bernal, Antonia Alcaraz, Joaquín Bravo, Ángeles Muñoz, Cristina Tomás, Eva Oliver, David Selva, Eva García, Román González, Elena Guijarro, Rodrigo Martínez, María Dolores Hernández.

**Hospital Universitario Clínico San Cecilio (Granada)**

Federico García, Clara Martínez, Leopoldo Muñoz Medina, Marta Álvarez, Natalia Chueca, David Vinuesa, Adolfo de Salazar, Ana Fuentes, Emilio Guirao, Laura Viñuela, Andrés Ruiz-Sancho, Francisco Anguita, Naya Faro, José Peregrina, Lucia Chaves, Marta Illescas.

**Centro Sanitario Sandoval (Madrid)**

Jorge Del Romero, Montserrat Raposo, Carmen Rodríguez, Teresa Puerta, Juan Carlos Carrió, Mar Vera, Juan Ballesteros, Oskar Ayerdi, Begoña Baza, Eva Orviz.

**Hospital Clínico Universitario de Santiago (Santiago de Compostela)**

Antonio Antela, Elena Losada.

**Hospital Universitario Son Espases (Palma de Mallorca)**

Melchor Riera, María Peñaranda, M Angels Ribas, Antoni A. Campins, Mercedes Garcia-Gazalla, Francisco J Fanjul, Javier Murillas, Francisco Homar, Helem H Vilchez, Luisa Martin, Antoni Payeras.

**Hospital Universitario Virgen de la Victoria (Málaga)**

Jesús Santos, María López, Crisitina Gómez, Isabel Viciana, Rosario Palacios.

**Hospital Universitario Virgen del Rocío (Sevilla)**

Luis Fernando López-Cortés, Nuria Espinosa, Cristina Roca, Silvia Llaves.

**Hospital Universitario de Bellvitge (Hospitalet de Llobregat)**

Juan Manuel Tiraboschi, Arkaitz Imaz, Ana Karina Silva, María Saumoy, Sofía Catalina Scévola.

**Hospital Universitario Valle de Hebrón (Barcelona)**

Adrián Curran, Vicenç Falcó, Jordi Navarro, Joaquin Burgos, Paula Suanzes, Jorge García, Vicente Descalzo, Patricia Álvarez, Bibiana Planas, Marta Sanchíz, Lucía Rodríguez, Arnau Monforte, Paola Vidovic.

**Hospital Costa del Sol (Marbella)**

Julián Olalla, Javier Pérez, Alfonso del Arco, Javier de la Torre, José Luis Prada.

**Hospital General Universitario Santa Lucía (Cartagena)**

Onofre Juan Martínez, Lorena Martinez, Francisco Jesús Vera, Josefina García, Begoña Alcaraz, Antonio Jesús Sánchez Guirao.

**Complejo Hospitalario Universitario a Coruña (CHUAC) (A Coruña)**

Álvaro Mena, Berta Pernas, Pilar Vázquez, Soledad López.

**Hospital Universitario Basurto (Bilbao)**

Sofía Ibarra, Guillermo García, Josu Mirena, Oscar Luis Ferrero, Josefina López, Mireia de la Peña, Miriam López, Iñigo López, Itxaso Lombide, Víctor Polo, Joana de Miguel, Beatriz Ruiz Estevez, Maite Ganchegui Aguirre, María Jesús Barberá Gracia.

**Hospital Universitario Virgen de la Arrixaca (El Palmar)**

Carlos Galera, Marian Fernández, Helena Albendin, Antonia Castillo, Asunción Iborra, Antonio Moreno, M Angustias Merlos, Inmaculada Chiclano.

**Hospital de la Marina Baixa (La Vila Joiosa)**

Concha Amador, Francisco Pasquau, Concepción Gil, José Tomás Algado.

**Hospital Universitario Infanta Sofía (San Sebastián de los Reyes)**

Inés Suarez-García, Eduardo Malmierca, Patricia González-Ruano, M Pilar Ruiz, José Francisco Pascual, Luz Balsalobre, Ángela Somodevilla.

**Hospital Universitario de Jaén (Jaén)**

María de la Villa López, Mohamed Omar, Carmen Herrero.

**Hospital Universitario San Agustín (Avilés)**

Miguel Alberto de Zarraga, Desiré Pérez.

**Hospital Clínico San Carlos (Madrid)**

Vicente Estrada, Noemí Cabello, M José Núñez, Iñigo Sagastagoitia, Reynaldo Homen, Ana Muñoz, Inés Armenteros Yeguas.

**Hospital Universitario Fundación Jiménez Díaz (Madrid)**

Miguel Górgolas, Alfonso Cabello, Beatriz Álvarez, Laura Prieto, Aws Al-Hayani, Irene Carrillo.

**Hospital Universitario Príncipe de Asturias (Alcalá de Henares)**

José Sanz, Alberto Arranz, Cristina Hernández, María Novella.

**Hospital Clínico Universitario de Valencia (Valencia)**

M José Galindo, Sandra Pérez Gómez, Ana Ferrer.

**Hospital Reina Sofía (Córdoba)**

Antonio Rivero Román, Inma Ruíz, Antonio Rivero Juárez, Pedro López, Isabel Machuca, Mario Frias, Ángela Camacho, Ignacio Pérez, Diana Corona, Javier Manuel Caballero.

**Hospital Universitario Severo Ochoa (Leganés)**

Miguel Cervero, Rafael Torres.

**Hospital Universitario Virgen de Valme (Sevilla)**

Juan Macías Sánchez, Pilar Rincón, Luis Miguel Real, Anais Corma, Alejandro Gonzalez-Serna.

**Hospital Álvaro Cunqueiro (Vigo)**

Eva Poveda, Alexandre Pérez, Luis Morano, Celia Miralles, Antonio Ocampo, Guillermo Pousada, María Gallego, Jacobo Alonso, Inés Martínez.

**Hospital Clínico Universitario de Valladolid (Valladolid)**

Carlos Dueñas, Sara Gutiérrez, Marta de la Fuente López, Cristina Novoa, Xjoylin Egües, Pablo Telleria.
